# *Sensors* 2020 Best Paper Awards

**DOI:** 10.3390/s20164355

**Published:** 2020-08-05

**Authors:** Sensors Editorial Office

**Affiliations:** MDPI AG, St. Alban-Anlage 66, 4052 Basel, Switzerland; sensors@mdpi.com

*Sensors* is instituting the Best Paper Award to recognize outstanding papers published in the journal. We are pleased to announce the “*Sensors* Best Paper Award” for 2020. Nominations were chosen from all papers published between 1 January 2018 and 31 December 2018. The Selection Committee examined each paper and expressed their preferences, releasing the ranking of the “*Sensors* 2020 Best Paper Award”. Following the review by the Editorial Board, the following top-voted research articles and review papers, in no particular order, have won a “*Sensors* 2020 Best Paper Award”.

## 1. Original Research Article Award

A Non-Destructive System Based on Electrical Tomography and Machine Learning to Analyze the Moisture of Buildings

by **Tomasz Rymarczyk, Grzegorz Kłosowski** and **Edward Kozłowski**

*Sensors***2018**, *18*, 2285, doi:10.3390/s18072285 

## 2. Review Paper Award

Recent Advances in Active Infrared Thermography for Non-Destructive Testing of Aerospace Components

by **Francesco Ciampa, Pooya Mahmoodi, Fulvio Pinto** and **Michele Meo**

*Sensors***2018**, *18*, 609; doi:10.3390/s18020609 

Trends Supporting the In-Field Use of Wearable Inertial Sensors for Sport Performance Evaluation: A Systematic Review

by **Valentina Camomilla, Elena Bergamini, Silvia Fantozzi** and **Giuseppe Vannozzi**

*Sensors***2018**, *18*, 873; doi:10.3390/s18030873 

These three outstanding papers are valuable contributions to *Sensors*. On behalf of the Editorial Board, we would like to congratulate these three teams for their excellent work. In recognition of their accomplishments, each team will receive a certificate and 500 CHF. We would like to take this opportunity to thank all the nominated research groups of the above exceptional papers for their contributions to *Sensors*, and thank the *Sensors* Editorial Board for voting and helping with this “Best Paper Award”.

